# Hepatocellular Carcinoma in Pakistan: National Trends and Global Perspective

**DOI:** 10.1155/2016/5942306

**Published:** 2016-02-03

**Authors:** Abu Bakar Hafeez Bhatti, Faisal Saud Dar, Anum Waheed, Kashif Shafique, Faisal Sultan, Najmul Hassan Shah

**Affiliations:** ^1^Department of HPB and Liver Transplantation, Shifa International Hospital, Islamabad, Pakistan; ^2^Sind Medical College, Karachi, Pakistan; ^3^Department of Public Health, Dow University of Health Sciences, Karachi, Pakistan; ^4^Department of Infectious Diseases, Shaukat Khanum Memorial Cancer Hospital and Research Center, Lahore, Pakistan; ^5^Department of Transplant Hepatology and Gastroenterology, Shifa International Hospital, Islamabad, Pakistan

## Abstract

Hepatocellular carcinoma (HCC) ranks second amongst all causes of cancer deaths globally. It is on a rise in Pakistan and might represent the most common cancer in adult males. Pakistan contributes significantly to global burden of hepatitis C, which is a known risk factor for HCC, and has one of the highest prevalence rates (>3%) in the world. In the absence of a national cancer registry and screening programs, prevalence of hepatitis and HCC only represents estimates of the real magnitude of this problem. In this review, we present various aspects of HCC in Pakistan, comparing and contrasting it with the global trends in cancer care. There is a general lack of awareness regarding risk factors of HCC in Pakistani population and prevalence of hepatitis C has increased. In addition, less common risk factors are also on a rise. Majority of patients present with advanced HCC and are not eligible for definitive treatment. We have attempted to highlight issues that have a significant bearing on HCC outcome in Pakistan. A set of strategies have been put forth that can potentially help reduce incidence and improve HCC outcome on national level.

## 1. Introduction

Cancer is a leading cause of death worldwide and accounted for 8.2 million deaths in the year 2012. Hepatocellular carcinoma (HCC) is the second most common cause of cancer death in the world with 745,000 deaths in the year 2012. In 2013, the World Health Organization (WHO) launched “Global Action Plan for the Prevention and Control of Noncommunicable Diseases 2013–2020.” The primary objective of this plan is to reduce premature mortality due to cancer, cardiovascular and respiratory disease, and diabetes by 25% [1].

Pakistan stands at the crossroads of socioeconomic insecurity and a keen desire for change [2]. It is the sixth most populous country in the world with estimated population of 182,142,594. As a low income country, we lag behind in various important determinants of healthcare when compared with peer countries. Based on WHO statistics, only 2.7% of total GDP is allocated to health sector (2012), infant mortality is 75.5 per 1000 live births (2012), maternal mortality is 170 per 100,000 live births (2013), and total health expenditure is 77$ per capita (2012). It is also estimated that 78% of our population pays out of pocket, private sector provides 3/4 of total healthcare, and there are twice as many doctors as nurses for our patients [3].

Cancer incidence and mortality are increasing in the developing world. Pakistan faces sinister limitations in cancer care that have an adverse impact on patient outcomes [4]. A steady increase in the incidence of hepatobiliary cancers has been observed. Based on results of a reliable hospital-based registry in Pakistan, hepatobiliary cancers are the most common malignancy in adult males and represent 10.7% of all cancers [5]. The age standardized rate for HCC in Pakistan is 7.6 per 100,000 persons per year for males and 2.8 for females [6–8]. Our knowledge on HCC in Pakistani population is limited and primarily reflects single center experiences. Data on HCC is derived from hepatitis B (hep-B) and hepatitis C (hep-C) patients and we are unaware of the natural history of non-hep-B/hep-C HCC in our population [9]. It is estimated that 60–70% HCC in Pakistan is attributable to hep-C. This is different from many other Asian Pacific countries where hep-B remains the predominant etiology [10–13]. Cancer care in developing countries is compromised but HCC is unique in a number of ways. It has geographical variation, treatment remains controversial, and resource intensive and technical competency alters the outcome considerably. Additionally, various obstacles limit access to standard healthcare and a delayed diagnosis unfortunately yields poor outcomes. Therefore applicability of established guidelines remains questionable in Pakistan and yet we lack a national consensus on HCC management.

Here, we have reviewed the epidemiology of HCC along with diagnostic interventions and treatments offered to Pakistani patients. Factors that lead to late presentation have also been discussed. We have elaborated upon the impact of emerging therapies on HCC in Pakistan and attempted to outline guidelines for better provision of care to our patients.

## 2. Risk Factors and Epidemiology 

Well known risk factors for HCC include alcohol consumption, chronic infection with hepatitis C and hepatitis B, autoimmune hepatitis, hereditary hemochromatosis, alpha-1 antitrypsin deficiency, Wilson disease, and porphyrias [16, 17]. There is increasing evidence that diabetes and obesity are linked to HCC [18–20].

### 2.1. Pakistan's Outlook

In the absence of a national registry for cancer patients, data primarily comes from single center experiences or scattered regional registries [6–8, 21, 22]. Most patients present in the fifth decade of life [23–25]. Hep-C is the most common etiology in up to 58% patients and hep-B in 25.3% cases [13]. Results from larger studies (*N* ≥ 100) on HCC are conflicting where anti-HCV antibody positivity ranges between 24 and 72.5% while HbsAg positivity varies between 13.1 and 51.2% [9, 26–31]. This can be attributed to patchy nature of available information, high prevalence of hep-C in certain regions of the country, and lack of national cancer surveillance. Analysis of molecular evolution points towards a distinct phylogenetic cluster of HCV-IIIa in our region around 1920s which was followed by a rapid exponential growth in 1950s. As a result, epidemic spread of HCV-IIIa occurred in Pakistan much earlier than other countries [11]. Factors implicated in spread of hep-C virus include a predominantly rural population (66%), illiteracy, unscreened blood products, and misuse of injectables [32]. Use of unsterilized instruments for shaving, minor surgical procedures, and circumcision is common in certain parts of Pakistan. It was shown that up to 48% barbers use unsterile blades for shaving [33]. This is probably why hep-B is the major factor responsible for HCC in developing Asian countries but not the most common etiology in Pakistan [34]. A rise in non-hep-B-hep-C HCC has also been observed. Underlying factors include rising incidence of diabetes, obesity, and aflatoxins [13, 35–37].

**Figure 1:**
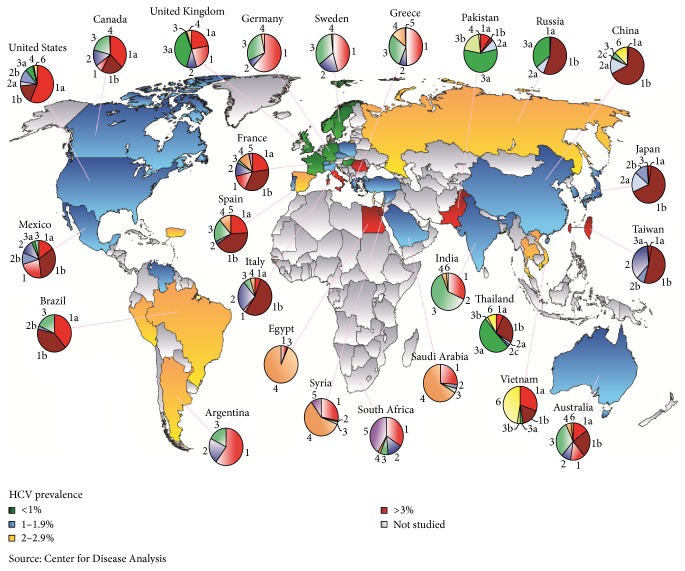
HCV prevalence among adults and genotype distribution.

To summarize, risk factors for HCC are not different in Pakistan than the rest of the world. Relative frequency of risk factors however is variable and hep-C is by far the most common etiology. As demonstrated in Figure 1, Pakistan is amongst the few countries in the world with the >3% prevalence of anti-HCV antibody. We remain unaware of the exact prevalence of non-hep-B-C HCC in Pakistan but increasingly sedentary life style, obesity, diabetes, and poor quality of food have an important role to play.

## 3. Incidence and Mortality 

Approximately 85% of global liver cancer burden is in Asia and Africa. China, Korea, and Japan are Asian countries with incidence greater than 20/100,000 population. In contrast, Northern Europe and North America are low incidence zones with incidence of HCC < 10/100,000 population.

### 3.1. Pakistan's Outlook

HCC is associated with male gender. It was shown that Pakistan is amongst few countries along with Zimbabwe, Columbia, and Costa Rica with no gender predilection but this might be changing now [34, 35]. Incidence of HCC in Pakistan is on a rise and correlates well with increasing exposure to risk factors for HCC in our population [10]. Recent results show that hepatobiliary cancers might represent the most common malignancy in adult males in our population [5]. Based on available data, age standardized rate for HCC in Pakistan is 7.6 per 100,000 persons per year for males and 2.8 for females [6–8]. These estimates are based on hospital-based data and do not reflect the true population-based prevalence of HCC in recent years.

## 4. Screening 

HCC has a median subclinical period of 3.2 years. In this period screening has the highest impact with early detection and potential for cure [38, 39]. Ultrasound (US) can detect tumors as small as 1.6 ± 0.6 cm [40]. Although US has sensitivity and specificity of >90% in detecting HCC, its efficiency is compromised in liver cirrhosis. Its yield largely depends on expertise of ultrasonographer [41].

Significant differences in regional guidelines on HCC screening and diagnosis exist. European association on study of liver-European organization for research and treatment of cancer (EASL-EORTC) recommends against the use of serum alpha fetoprotein (AFP) for regular screening given its low specificity and additional cost per primary liver cancer detected ($1982 (US alone) versus $3639 (AFP + US)) [15, 42–44]. Asian Oncology Summit (AOS) recommends 3–6 monthly US with serum AFP. AOS guidelines are the least stringent given the high incidence of risk factors (hep-B and hep-C) and HCC in this region. In addition AOS recommends an AFP > 400 ng/mL to be diagnostic for HCC in high risk patients [45, 46].

### 4.1. Pakistan's Outlook

Majority of patients with risk factors for HCC do not undergo screening. We do not have nationally accepted guidelines for screening high risk patients and physicians at large have a variable practice in terms of choice of investigations and time period between them. The most common trend is 6 monthly US and serum AFP level [47]. Many a time, screening US is performed by inexperienced sonographers. Background cirrhosis makes interpretation of US findings difficult. Less than 10% patients are diagnosed with HCC on screening in Pakistan [9, 13] and that perhaps explains the delayed presentation and poor prognosis in majority of HCC patients. The association between elevated AFP and HCC diagnosis is variable. HCC might be present in 7.5% to 100% patients with raised AFP [13, 27, 48–50]. This heterogeneity primarily stems from variable cut-offs used to define elevated AFP levels.

In summary, as low as 10% patients in Pakistan with risk factors for HCC undergo regular screening and majority of patients are diagnosed when they are symptomatic. For those who are screened; US and AFP are the most frequently performed investigations but the time duration between these investigations, cut-off for elevated AFP, and sonographers' technical competency remain grey areas. Recently, Pakistan society for study of liver disease (PSSLD) has recommended 6 monthly US for screening in cirrhotic patients.

## 5. Diagnosis and Staging

Diagnostic criteria in HCC remain controversial. This is particularly true for lesions < 1 cm in size. For lesions > 1 cm in size, typical features on CT or MRI are sufficient for establishing a diagnosis. National comprehensive cancer network (NCCN) and EASL-EORTC recommend 4–6 monthly surveillance with US, CT, or MRI for lesions < 1 cm in size if they do not exhibit typical arterial enhancement and venous washout on CT or MRI. For lesions > 1 cm but atypical features on imaging, biopsy is recommended if it is likely to alter management [15, 41]. AOS however recommends considering < 1 cm lesions with typical characteristics as HCC. AOS guidelines seem more applicable to Asian continent where HCC is more common, hepatitis B and hepatitis C are prevalent, and appropriate surveillance is difficult. In addition AFP > 400 is also diagnostic for HCC. Biopsy is recommended in patients with a doubtful diagnosis [45, 46].

According to recently concluded BRIDGE study, the most common stage at presentation for patients with HCC remains BCLC stage C except in Japan and Taiwan [14]. Both these countries have initiated national surveillance programs which are still lacking in North America, Europe, and China [51–54].

### 5.1. Pakistan's Outlook

Only 1.7% patients are diagnosed on CT findings alone while various combinations of CT, AFP, and histopathology are used in 62.5% patients [28]. Late presentation and advanced cirrhosis in majority patients are contributory. Since fewer than 10% patients are picked up on screening in Pakistan, patients usually have large tumors (≥8 cm) at the time of diagnosis. Tumors larger than 5 cm are seen in 44.3% patients and at presentation; 52–62% patients have more than 1 tumor nodule. In addition 46–87% patients have Child-Pugh stage B or C. Around 86% patients belong to Okuda class II or III and less than 15% patients are amenable to any form of definitive treatment [9, 28].

To summarize, patients with HCC generally have advanced disease at presentation and very few of them are eligible for definitive treatment. Since no local guidelines exist for diagnosis of HCC, majority of patients end up undergoing an array of expensive investigations for establishing a diagnosis.

## 6. Treatment and Survival 

HCC has a poor prognosis even in developed countries and 5-year survival is only 10%. Survival is even worse in developing countries and mortality is roughly equivalent to incidence rates [16]. According to 2015 statistics of International Agency for Research on Cancer (IARC), the mortality to incidence ratio for HCC is 0.95 and geographical patterns of incidence to mortality are nearly uniform [55]. Early detection of HCC is critical in ensuring optimal treatment. Tumor characteristics (size, multinodularity, and vascular invasion), underlying liver function (Child-Pugh score) and performance status (Eastern Cooperative Oncology Group performance status), play an important role in survival [56–58].

**Figure 2:**
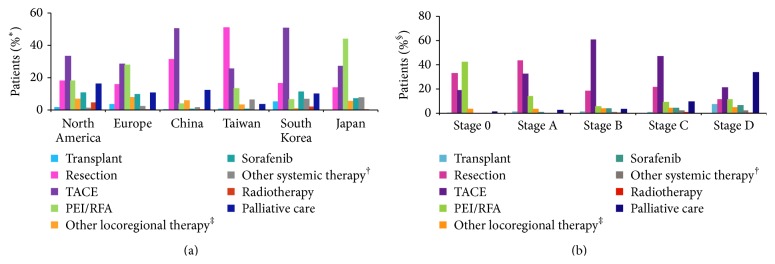
Treatment for HCC stratified based on region and stage. (From [14]).

Since prognosis of HCC depends on multiple factors, various algorithms and guidelines have been adopted but have failed to satisfactorily address issues in HCC management. The most widely used algorithm is Barcelona Clinic Liver Cancer (BCLC) staging system. It incorporates tumor characteristics, liver function, and performance status of an individual patient for allocation to different stages. Patients in stages 0 and A are eligible for potentially curative treatment options like surgical resection, transplantation, and local ablation. For patients in stage B, transarterial chemoembolization (TACE) is utilized. Stage C patients are treated with Sorafenib while stage D (terminal) patients are managed supportively [59–61]. Global trends in HCC treatment are not uniform and are dictated by data collection instruments, availability of treatment facilities, and technical skills. Treatment may vary for the same stage across different regions. TACE is the most frequently used first treatment in North America, Europe, China, and South Korea; PEI/RFA in Japan; and resection in Taiwan (Figure 2) [14].

Since 25–70% patients with HCC have advanced stage at presentation [62–66], chemotherapy provides minimal benefit and survival with Sorafenib does not extend beyond a median of 2-3 months [67, 68]. Based on results of clinical trials, median overall survival (OS) is 20 months for stage B HCC, 10 months for Stage C HCC treated with Sorafenib, and 3 months for stage D [67, 69]. It must be noted that these guidelines are not applicable to all patients and treatment decisions for individual patients should ideally be personalized. Given the complexity of disease, decisions for individual patients should be taken by multidisciplinary teams [70–72].

### 6.1. Pakistan's Outlook

Majority of patients in Pakistan only receive supportive care due to advanced stage at presentation. Yusuf and colleagues reviewed outcomes of 584 patients seen in a cancer hospital in Pakistan. Only 79 (13.5%) received definitive treatment while the rest were managed with supportive care [28]. TACE was the most frequent treatment administered in 60.7% of these 79 patients followed by PEI in 21.5% and resection in 17.7% patients. The cumulative probability of survival was 45%, 20%, and 10% at 1, 3, and 5 years. Another study reported outcomes in 645 HCC patients. Again TACE was the most commonly used treatment in 38.2% patients. All patients had BCLC stage B. Only 2.8% patients in this study had BCLC stage 0 or A HCC [9]. It is important to know that these results come from tertiary care hospitals which are well equipped with medical resources. Majority of patients with HCC never reach specialist facility and we are unaware of their outcomes. Although multidisciplinary teams have recently become more popular in Pakistan, even then the majority of patients are being treated by physicians alone and a multidisciplinary input still remains lacking [73].

## 7. Research on HCC

**Figure 3:**
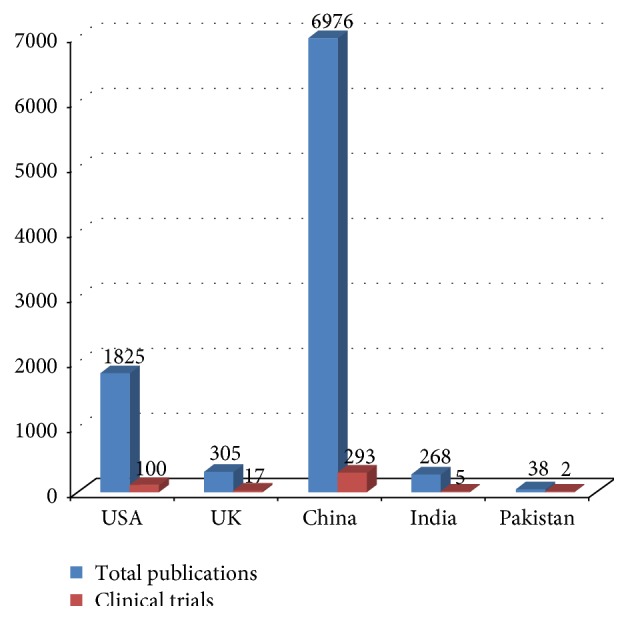
Research on HCC in last 25 years (retrieved from PubMed on 30 April 2015).

Globally, research on HCC lags behind certain other cancers, for example, breast. There is paucity of randomized trials and most studies are retrospective clinical observations. In the last 25 years, there have been 46,959 publications on breast cancer from the United States including 3097 clinical trials [74]. We attempted to assess Pakistan's contribution to HCC research in the last 25 years. PubMed was searched for publications related to HCC from USA, UK, China, India, and Pakistan. As shown in Figure 3, China is the major contributor for HCC research. A total of 6976 publications were retrieved from China versus 2436 from all other countries. Contribution in terms of clinical trials was also higher in China as compared to all other countries, that is, 293 versus 124, respectively.

### 7.1. Pakistan's Outlook

In the last 15 years, 38 publications including 2 clinical trials were conducted in Pakistan. Both these trials assessed outcomes of advanced HCC with sorafenib/gemcitabine or arterial infusion of ifosfamide [75, 76]. At present, Pakistan is participating in 4 multicenter HCC clinical trials [77]. The scarcity of data on HCC reflects upon the advanced stage at presentation when most treatment strategies are futile. Limitations in availability of treatment facilities in Pakistan are a contributing factor [13]. Very few centers in Pakistan offer surgical resection for HCC and liver transplantation was not offered at all until recently [78, 79]. Treatment facilities for PEI, RFA, and TACE are inadequate and are available predominantly to affording patients who only represent a minor fraction of patients suffering from HCC in Pakistan.

## 8. Discussion

### 8.1. National Screening Program

Majority of HCC in Pakistan is hep-C related. Even today, we remain unaware of the exact prevalence of hep-C in Pakistan. It is assumed that around 10 million people in Pakistan are infected with hep-C [80, 81]. It is imperative to have a national hep-C and hep-B screening program. This would allow correct estimates of disease burden in our population and present true picture of viral hepatitis. It has been shown that patients diagnosed with HCC during surveillance have less advanced disease, are more likely to be eligible for curative treatment, and are likely to have increased survival [82].  Table 1 represents various obstacles and probable solutions for HCC management in Pakistan.

### 8.2. Public Education

**Figure 4:**
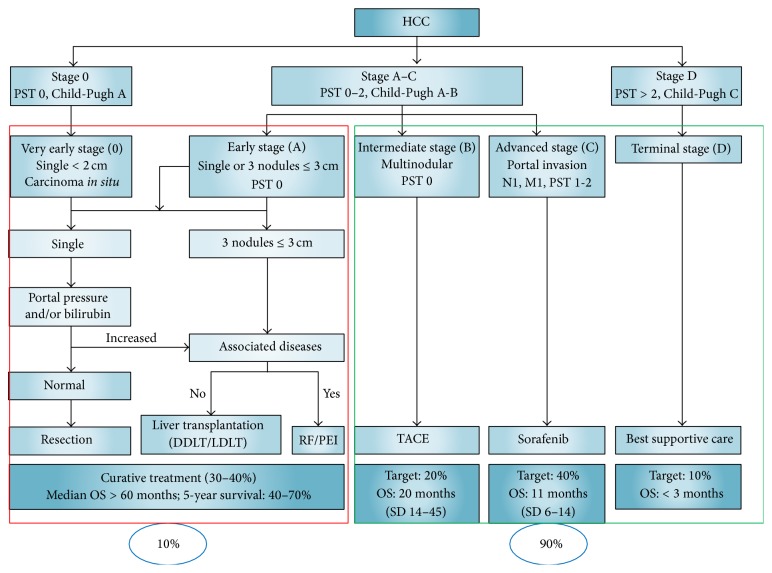
BCLC staging and relative distribution of Pakistani patients at the time of presentation. (Adapted from [15]).

Hepatitis C and hepatitis B are both preventable. People in Pakistan generally remain unaware of risk factors and need to be educated regarding modes of spread and necessary precautions [83, 84]. Appropriate blood product screening, prohibition of reuse of syringes, sterilization of instruments for circumcision, dental procedures, hair cutting, and shaving may tremendously reduce the incidence of hep-C and hep-B infection [85]. Audiovisual dissemination of risk factors for hepatitis and its impact on health outcomes needs to be communicated to the public. In addition patients infected with hepatitis should understand benefits of strict surveillance and treatments available for HCC.  Figure 4 demonstrates the BCLC staging algorithm. Based on results from Pakistan, only 10% patients present in early stage and have the potential to be cured. Unfortunately 90% patients present in advanced stage and are not candidates for curative therapy. Our short term goal should be the development of palliative care facilities and better provision of TACE and Sorafenib to patients with advanced HCC. Indeed palliative care is one of the most ignored specialties in Pakistan. In the long term, we need to develop facilities to detect HCC early and increase patient pool eligible for curative therapy. Physician and public awareness regarding risk factors for HCC, screening in the presence of risk factors, and information on treatments available (what and where) in Pakistan is required.

### 8.3. Diabetes and Obesity

According to International Diabetes Federation, Pakistan is among the leading countries with high prevalence of DM and more than 20% population is suffering from diabetes [86, 87]. As high as 50% patients with non-hep-B/C HCC have diabetes in Pakistan [9]. It has been shown that patients with HCC and diabetes treated with Sorafenib and metformin have poor outcomes compared with patients with HCC alone due to tumor aggressiveness in the diabetic group [88]. Sedentary life style has contributed enormously. General public needs to be educated regarding association of obesity/diabetes with multiple cancers and inclusion of healthy life style and balanced diet in daily life.

### 8.4. Development of Infrastructure

Only a handful of centers in Pakistan provide facilities for TACE, RFA, and PEI. As HCC incidence rises, the demand and supply gap will widen significantly. At least one center in every major city should be planned. This can take pressure off from the more specialized centers and long waiting times for treatment can be avoided.

### 8.5. Acquire Technical Skills

Surgical resection for early stage HCC is performed only in a few centers in Pakistan. Liver transplantation was not offered until 2012, but it is offered now in Islamabad and Lahore. Overall, surgical care for HCC patients is compromised and there is lack of technical skills as specialized HPB centers are emerging but are yet not established. Technical skills for TACE, RFA, and PEI are also restricted to a few centers and population at large is not being benefited. Public sector needs to come forward and attempt to acquire skills and facilities so that they can be provided to community at large.

### 8.6. Communication, Collective Decision Making

HCC is a difficult disease to manage. There is an array of staging algorithms and wide variations exist in the use of treatment modalities. HCC management should be performed collectively by multidisciplinary teams and personalized approach to patient care should be idealized. For physicians managing HCC patients in suburban/rural regions, an online MDT can be established to aid better decision making.

### 8.7. New Treatments

After years of research, we are facing an interesting period where new drugs for hepatitis C will be approved every year. In late 2013, Sofosbuvir was approved by FDA in USA and European Medicine Agency in early 2014. In a phase III randomized trial, Sofosbuvir and Ledipasvir have shown a sustained viral response of >96% [89]. A 12-week course of Sofosbuvir can cost up to 84000 US dollars [90]. The cost has great implications in terms of its availability in low income countries. In 2006, Egypt developed highly specialized centers for treatment of hepatitis C and brought the cost of Peg-interferon and ribavirin down to <10% of its cost in USA via successful negotiations with the pharmaceutical companies. Within 6 years of this program, 300,000 Egyptians were treated [91]. Fortunately, similar negotiations have resulted in a reduction in cost of Sofosbuvir for South Asian patients. It is available in 3000 dollars at appointed places in Pakistan. A sustained commitment from Pakistani government can ensure easy availability of these drugs to treat more patients. More transplant centers need to be developed as transplant provides one of the best forms of definitive treatment for patients with HCC. Internationally there is a growing debate over expansion of donor criteria for liver transplant [92]. Better systemic chemotherapy, targeted agents, and immune therapy need to be developed due to limited impact of existing systemic therapies [93]. Various genetic and molecular pathways have been explored. It has been suggested that understanding of epigenetic and genetic processes in HCC might help overcome therapeutic stranding in HCC [94].

### 8.8. Training Future Caregivers

At present, very few physicians return to Pakistan after receiving advanced training in other countries. Local doctors interested in treatment of patients with liver diseases should be contracted for training and offered viable positions to ensure adequate numbers of healthcare personnel in the country.

### 8.9. National Cancer Registry

It is high time that a national cancer registry is developed. We need to have correct estimates of healthcare burden in general and cancer in particular in our population. It is difficult to develop a national policy/guideline based on results of scattered registries or hospital data. The true incidence and prevalence of various cancers needs to be registered so that a national action plan can be developed accordingly.

## 9. Conclusion

HCC in Pakistan is likely to consume significant hospital resources and drain a sizeable chunk of health budget in the future. Although HCC is a multifaceted disease, it largely remains preventable. Surprisingly, general population remains unaware of simple measures that can drastically reduce its occurrence. A balanced approach is required in trying to combat HCC. Prevention, surveillance, and appropriate treatment can significantly improve outcomes and decrease incidence of HCC in Pakistan.

## Figures and Tables

**Table 1:** Guidelines for HCC management in Pakistan.

Issues	Potential solutions
Incidence and prevalence of hepatitis C and hepatitis B are unknown	Implementation of national screening program

Lack of awareness regarding risk factors Low screening rate High prevalence of diabetes and obesity	Public education via audiovisual dissemination regarding risk factor prevention, maintenance of healthy life style, and exercise

Insufficient HPB/liver transplant, palliative care, and cancer centers Enormous demand and supply gap	Development of infrastructure with collaboration of public and private sector

Shortage of technical skills	Acquire technical skills via international exposure and collaboration

Controversial aspects of HCC management Knowledge gap	Collective decision making Online tumor boards Personalized care for individual patients

Low remission rates for hepatitis C	Access to new treatments at affordable price

Physician shortage	Train future caregivers Incentivization

Scattered cancer registries True cancer incidence and prevalence unknown	National cancer registry

## References

[1] IARC (2014). *World Cancer Report 2014*.

[2] Nishtar S., Bhutta Z. A., Jafar T. H. (2013). Health reform in Pakistan: a call to action. *The Lancet*.

[3] Nishtar S., Boerma T., Amjad S. (2013). Pakistan's health system: performance and prospects after the 18th Constitutional Amendment. *The Lancet*.

[4] Lyerly H. K., Fawzy M. R., Aziz Z. (2015). Regional variation in identified cancer care needs of early-career oncologists in China, India, and Pakistan. *The Oncologist*.

[5] Badar F., Mahmood S. (2015). Hospital-based cancer profile at the Shaukat Khanum Memorial Cancer Hospital and Research Centre, Lahore, Pakistan. *Journal of the College of Physicians and Surgeons Pakistan*.

[6] Bhurgri Y., Bhurgri A., Hassan S. H. (2000). Cancer incidence in Karachi, Pakistan: first results from Karachi Cancer Registry. *International Journal of Cancer*.

[7] Bhurgri Y., Bhurgri A., Pervez S. (1998). Cancer profile of hyderabad, Pakistan 1998–2002. *Asian Pacific Journal of Cancer Prevention*.

[8] Bhurgri Y., Pervez S., Kayani N. (2006). Cancer profile of Larkana, Pakistan (2000–2002). *Asian Pacific Journal of Cancer Prevention*.

[9] Butt A. S., Hamid S., Wadalawala A. A. (2013). Hepatocellular carcinoma in Native South Asian Pakistani population; Trends, clinico-pathological characteristics & differences in viral marker negative & viral-hepatocellular carcinoma. *BMC Research Notes*.

[10] Abbas Z. (2013). Hepatocellular carcinoma in Pakistan. *Journal of the College of Physicians and Surgeons Pakistan*.

[11] Khan A., Tanaka Y., Azam Z. (2009). Epidemic spread of hepatitis C virus genotype 3a and relation to high incidence of hepatocellular carcinoma in Pakistan. *Journal of Medical Virology*.

[12] Idrees M., Rafique S., Rehman I.-U. (2009). Hepatitis C virus genotype 3a infection and hepatocellular carcinoma: Pakistan experience. *World Journal of Gastroenterology*.

[13] Butt A. S., Abbas Z., Jafri W. (2012). Hepatocellular carcinoma in pakistan: where do we stand?. *Hepatitis Monthly*.

[14] Park J. W., Chen M., Colombo M. (2015). Global patterns of hepatocellular carcinoma management from diagnosis to death: the BRIDGE Study. *Liver International*.

[15] European Association for the Study of the Liver (2012). EASL-EORTC clinical practice guidelines: management of hepatocellular carcinoma. *Journal of Hepatology*.

[16] McGlynn K. A., London W. T. (2011). The global epidemiology of hepatocellular carcinoma: present and future. *Clinics in Liver Disease*.

[17] Calle E. E., Teras L. R., Thun M. J. (2005). Obesity and mortality. *The New England Journal of Medicine*.

[18] Welzel T. M., Graubard B. I., Quraishi S. (2013). Population-attributable fractions of risk factors for hepatocellular carcinoma in the United States. *American Journal of Gastroenterology*.

[19] El-Serag H. B., Hampel H., Javadi F. (2006). The association between diabetes and hepatocellular carcinoma: a systematic review of epidemiologic evidence. *Clinical Gastroenterology and Hepatology*.

[20] Wang C., Wang X., Gong G. (2012). Increased risk of hepatocellular carcinoma in patients with diabetes mellitus: a systematic review and meta-analysis of cohort studies. *International Journal of Cancer*.

[21] Bhurgri Y. (2004). Karachi cancer registry data—implications for the national cancer control program of Pakistan. *Asian Pacific Journal of Cancer Prevention*.

[22] Bhurgri Y., Pervez S., Usman A. (2002). Cancer patterns in Quetta (1998-1999). *The Journal of the Pakistan Medical Association*.

[23] Chohan A. R., Umar M., Khar B. (2001). Demographic features of hepatocellular carcinoma: a study of 30 cases. *Journal of Rawalpindi Medical College*.

[24] Butt A. K., Khan A. A., Alam A. (1998). Hepatocellular carcinoma: analysis of 76 cases. *Journal of the Pakistan Medical Association*.

[25] Gill M. L., Atiq M., Sattar S., Khokhar N. (2005). Treatment outcomes with long acting octreotide in inoperable hepatocellular carcinoma: a local experience and review of literature. *Journal of the Pakistan Medical Association*.

[26] Munaf A., Memon M. S., Kumar P., Ahmed S., Kumar M. B. (2014). Comparison of viral hepatitis-associated hepatocellular carcinoma due to HBV and HCV—cohort from liver clinics in Pakistan. *Asian Pacific Journal of Cancer Prevention*.

[27] Sharieff S., Burney I., Salam A., Siddiqui T. (2002). Hepatocellular carcinoma. *Journal of the College of Physicians and Surgeons Pakistan*.

[28] Yusuf M. A., Badar F., Meerza F. (2007). Survival from hepatocellular carcinoma at a cancer hospital in Pakistan. *Asian Pacific Journal of Cancer Prevention*.

[29] Abbas Z., Siddiqui A.-U., Luck N. H. (2008). Prognostic factors of survival in patients with non-resectable hepatocellular carcinoma: hepatitis C versus miscellaneous etiology. *Journal of the Pakistan Medical Association*.

[30] Ali R., Iqbal K., Irfan J. (2010). Characteristics of hepatocellular carcinoma patients attending NORI. *Annals of Pakistan Institute of Medical Sciences*.

[31] Ansari S., Memon M. S., Devrajani B. R. (2009). Frequency of hepatitis B and hepatitis C in patients with hepatocellular carcinoma at hyderabad. *Journal of the Liaquat University of Medical & Health Sciences*.

[32] Abbas Z., Jeswani N. L., Kakepoto G. N., Islam M., Mehdi K., Jafri W. (2008). Prevalence and mode of spread of hepatitis B and C in rural Sindh, Pakistan. *Tropical Gastroenterology*.

[33] Wazir M. S., Mehmood S., Ahmed A., Jadoon H. R. (2008). Awareness among barbers about health hazards associated with their profession. *Journal of Ayub Medical College, Abbottabad*.

[34] Fan J.-H., Wang J.-B., Jiang Y. (2013). Attributable causes of liver cancer mortality and incidence in China. *Asian Pacific Journal of Cancer Prevention*.

[35] Liu Y., Wu F. (2010). Global burden of Aflatoxin-induced hepatocellular carcinoma: a risk assessment. *Environmental Health Perspectives*.

[36] Nizami H. M., Zuberi S. J. (1977). Aflatoxin and liver cancer in Karachi, a preliminary survey. *Journal of the Pakistan Medical Association*.

[37] Iqbal Q., Amjad M., Asi M. R., Arino A. (2011). Assessment of hot peppers for aflatoxin and mold proliferation during storage. *Journal of Food Protection*.

[38] Barbara L., Benzi G., Gaiani S. (1992). Natural history of small untreated hepatocellular carcinoma in cirrhosis: a multivariate analysis of prognostic factors of tumor growth rate and patient survival. *Hepatology*.

[39] Sheu J.-C., Sung J.-L., Chen D.-S. (1985). Growth rate of asymptomatic hepatocellular carcinoma and its clinical implications. *Gastroenterology*.

[40] Sato T., Tateishi R., Yoshida H. (2009). Ultrasound surveillance for early detection of hepatocellular carcinoma among patients with chronic hepatitis C. *Hepatology International*.

[41] National Comprehensive Cancer Network (NCCN) (2013). *NCCN Clinical Practice Guidelines in Oncology: Hepatobiliary Cancers. Version 2013*.

[42] Villanueva A., Minguez B., Forner A., Reig M., Llovet J. M. (2010). Hepatocellular carcinoma: novel molecular approaches for diagnosis, prognosis, and therapy. *Annual Review of Medicine*.

[43] Hoshida Y., Nijman S. M. B., Kobayashi M. (2009). Integrative transcriptome analysis reveals common molecular subclasses of human hepatocellular carcinoma. *Cancer Research*.

[44] Zhang B., Yang B. (1999). Combined *α* fetoprotein testing and ultrasonography as a screening test for primary liver cancer. *Journal of Medical Screening*.

[45] Poon D., Anderson B. O., Chen L.-T. (2009). Management of hepatocellular carcinoma in Asia: consensus statement from the Asian Oncology Summit 2009. *The Lancet Oncology*.

[46] Parikh P., Malhotra H., Jelic S. (2008). EMSO Guidelines Working Group. Hepatocellular carcinoma: ESMO clinical recommendations for diagnosis, treatment and follow-up. *Annals of Oncology*.

[47] Phulpoto J. A., Shah I. A., Bhatti Z. (2012). Prevalence of hepatocellular carcinoma in cirrhotic patients of Northern Sindh attending liver clinics at Ghulam Mohammad Mahar Medical College Hospitals Sukkur and Khairpur. *Journal of the Liaquat University of Medical and Health Sciences*.

[48] Baig J. A., Alam J. M., Mahmood S. R. (2009). Hepatocellular carcinoma (HCC) and diagnostic significance of A-fetoprotein (AFP). *Journal of Ayub Medical College, Abbottabad*.

[49] Khokhar N., Aijazi I., Gill M. L. (2003). Spectrum of hepatocellular carcinoma at Shifa International Hospital, Islamabad. *Journal of Ayub Medical College, Abbottabad*.

[50] Zia ud Din, Muhammad R., Saeedi M. I., Mahmood K. (2006). Frequency of hepatoma in hepatitis B and C positive cirrhotic patients. *Journal of Postgraduate Medical Institute*.

[51] Chen C.-J., You S.-L., Lin L.-H., Hsu W.-L., Yang Y.-W. (2002). Cancer epidemiology and control in Taiwan: a brief review. *Japanese Journal of Clinical Oncology*.

[52] Yoo K. Y. (2008). Cancer control activities in the Republic of Korea. *Japanese Journal of Clinical Oncology*.

[53] Kudo M., Han K. H., Kokudo N. (2010). Liver cancer working group report. *Japanese Journal of Clinical Oncology*.

[54] Song P., Gao J., Inagaki Y. (2013). Biomarkers: evaluation of screening for and early diagnosis of hepatocellular carcinoma in Japan and china. *Liver Cancer*.

[55] Ferlay J., Parkin D. M., Curado M. P. (2007). *Cancer Incidence in Five Continents, Volumes I to IX*.

[56] Okuda K., Ohtsuki T., Obata H. (1985). Natural history of hepatocellular carcinoma and prognosis in relation to treatment. Study of 850 patients. *Cancer*.

[57] Llovet J. M., Bustamante J., Castells A. (1999). Natural history of untreated nonsurgical hepatocellular carcinoma: rationale for the design and evaluation of therapeutic trials. *Hepatology*.

[58] Cabibbo G., Enea M., Attanasio M., Bruix J., Craxí A., Cammà C. (2010). A meta-analysis of survival rates of untreated patients in randomized clinical trials of hepatocellular carcinoma. *Hepatology*.

[59] Cillo U., Bassanello M., Vitale A. (2004). The critical issue of hepatocellular carcinoma prognostic classification: which is the best tool available?. *Journal of Hepatology*.

[60] Cillo U., Vitale A., Grigoletto F. (2006). Prospective validation of the Barcelona Clinic Liver Cancer staging system. *Journal of Hepatology*.

[61] Wang J.-H., Changchien C.-S., Hu T.-H. (2008). The efficacy of treatment schedules according to Barcelona Clinic Liver Cancer staging for hepatocellular carcinoma—survival analysis of 3892 patients. *European Journal of Cancer*.

[62] Llovet J. M., Brú C., Bruix J. (1999). Prognosis of hepatocellular carcinoma: the BCLC staging classification. *Seminars in Liver Disease*.

[63] Altekruse S. F., McGlynn K. A., Reichman M. E. (2009). Hepatocellular carcinoma incidence, mortality, and survival trends in the United States from 1975 to 2005. *Journal of Clinical Oncology*.

[64] Howlader N., Noone A. M., Krapcho M. (2013). *SEER Cancer Statistics Review, 1975–2010*.

[65] Sloane D., Chen H., Howell C. (2006). Racial disparity in primary hepatocellular carcinoma: tumor stage at presentation, surgical treatment and survival. *Journal of the National Medical Association*.

[66] Carrilho F. J., Kikuchi L., Branco F., Gonçalves C. S., de Mattos A. A. (2010). Clinical and epidemiological aspects of hepatocellular carcinoma in Brazil. *Clinics*.

[67] Thomas M. B., Jaffe D., Choti M. M. (2010). Hepatocellular carcinoma: consensus recommendations of the National Cancer Institute Clinical Trials Planning Meeting. *Journal of Clinical Oncology*.

[68] Llovet J. M., Ricci S., Mazzaferro V. (2008). Sorafenib in advanced hepatocellular carcinoma. *The New England Journal of Medicine*.

[69] Cheng A.-L., Kang Y.-K., Chen Z. (2009). Efficacy and safety of sorafenib in patients in the Asia-Pacific region with advanced hepatocellular carcinoma: a phase III randomised, double-blind, placebo-controlled trial. *The Lancet Oncology*.

[70] Llovet J. M., Di Bisceglie A. M., Bruix J. (2008). Panel of Experts in HCC-Design Clinical Trials. Design and endpoints of clinical trials in hepatocellular carcinoma. *Journal of the National Cancer Institute*.

[71] Colombo M., Raoul J.-L., Lencioni R. (2013). Multidisciplinary strategies to improve treatment outcomes in hepatocellular carcinoma: a European perspective. *European Journal of Gastroenterology and Hepatology*.

[72] Roayaie S., Frischer J. S., Emre S. H. (2002). Long-term results with multimodal adjuvant therapy and liver transplantation for the treatment of hepatocellular carcinomas larger than 5 centimeters. *Annals of Surgery*.

[73] Junaid Nazar C. M., Kindratt T. B., Ahmad S. M., Ahmed M., Anderson J. (2014). Barriers to the successful practice of chronic kidney diseases at the primary health care level; A systematic review. *Journal of Renal Injury Prevention*.

[74] http://globocan.iarc.fr/Pages/fact_sheets_cancer.aspx.

[75] Naqi N., Ahmad S., Murad S., Khattak J. (2014). Efficacy and safety of sorafenib-gemcitabine combination therapy in advanced hepatocellular carcinoma: an open-label phase II feasibility study. *Hematology/Oncology and Stem Cell Therapy*.

[76] Malik I. A., Khan W. A., Haq S., Sabih M. (1997). A prospective Phase II trial to evaluate the efficacy and toxicity of hepatic arterial infusion of ifosfamide in patients with inoperable localized hepatocellular carcinoma. *American Journal of Clinical Oncology: Cancer Clinical Trials*.

[77] https://clinicaltrials.gov/ct2/results?term=Hepatocellular+carcinoma+Pakistan+&Search=Search.

[78] Bhatti A. B., Zia H., Dar F. S. (2015). Quality of life after living donor hepatectomy for liver transplantation. *World Journal of Surgery*.

[79] Dar F. S., Bhatti A. B., Dogar A. W. (2015). The travails of setting up a living donor liver transplant program: first program experience from Pakistan and lessons learned. *Liver Transplantation*.

[80] Jamil M. S., Ali H., Shaheen R., Basit A. (2010). Prevalence, knowledge and awareness of hepatitis C among residents of three Union Councils in Mansehra. *Journal of Ayub Medical College, Abbottabad*.

[81] Raja N. S., Janjua K. A. (2008). Epidemiology of hepatitis C virus infection in Pakistan. *Journal of Microbiology, Immunology and Infection*.

[82] Yang J. D., Harmsen W. S., Slettedahl S. W. (2011). Factors that affect risk for hepatocellular carcinoma and effects of surveillance. *Clinical Gastroenterology and Hepatology*.

[83] Altaf A., Shah S. A., Shaikh K., Constable F. M., Khamassi S. (2013). Lessons learned from a community based intervention to improve injection safety in Pakistan. *BMC Research Notes*.

[84] Faiz-Ur-Rehman Khan J., Fida Z., Parvez A., Rafiq A., Syed S. (2011). Identifiable risk factors in hepatitis B and C. *Journal of Ayub Medical College Abbottabad*.

[85] Ali S. A., Donahue R. M. J., Qureshi H., Vermund S. H. (2009). Hepatitis B and hepatitis C in Pakistan: prevalence and risk factors. *International Journal of Infectious Diseases*.

[86] http://www.idf.org/Diabetesatlas/Data-Visualisations.

[87] Zahid N., Claussen B., Hussain A. (2008). Diabetes and impaired glucose tolerance in a rural area in Pakistan and associated risk factors. *Diabetes and Metabolic Syndrome: Clinical Research and Reviews*.

[88] Casadei Gardini A., Marisi G., Scarpi E. (2015). Effects of metformin on clinical outcome in diabetic patients with advanced HCC receiving sorafenib. *Expert Opinion on Pharmacotherapy*.

[89] Wei L., Lok A. S. F. (2014). Impact of new hepatitis c treatments in different regions of the world. *Gastroenterology*.

[90] Knox R. $1,000 pill for hepatitis C spurs debate over drug prices. http://www.npr.org/blogs/health.

[91] Pawlotsky J. M. An interview with Professor Jean-Michel Pawlotskyon how the treatment of HCV in-fection will change in the nearfuture as a result of recent developments. http://www.hepbcppa.org/newsletter.

[92] Waller L. P., Deshpande V., Pyrsopoulos N. (2015). Hepatocellular carcinoma: a comprehensive review. *World Journal of Hepatology*.

[93] Ge S., Huang D. (2015). Systemic therapies for hepatocellular carcinoma. *Drug Discoveries & Therapeutics*.

[94] Gnoni A., Santini D., Scartozzi M. (2015). Hepatocellular carcinoma treatment over sorafenib: epigenetics, microRNAs and microenvironment. Is there a light at the end of the tunnel?. *Expert Opinion on Therapeutic Targets*.

